# The Severer Forms of Laryngitis

**Published:** 1920-03-27

**Authors:** 


### THE SEVERER FORMS OF LARYNGITIS.

### II. Early Diagnosis and Treatment (*continued*).

5. Acute Septic Laryngitis.

The different forms of acute septic inflammation of the mouth and faucial regions, acute œdema, phlegmon, and erysipelas of the pharynx and larynx, together with cellulitis of the submaxillary region (Ludovic's angina) are probably identical diseases, only varying, pathologically, in their degrees of virulence.

This laryngeal lesion appears to be but rarely primary in its origin, but is generally secondarily produced by direct or indirect extensions from lesions in the neighbourhood, and in every case a careful search should be made to determine the site of the primary infection.

*Symptoms and Signs—Early.*—Often begin with pseudo or genuine rigors; there is high fever, and in very severe cases hyperpyrexia is not uncommon; the pulse, at first, is full and bounding, but soon becomes rapid and feeble, the patient passing into a state of asthenia and delirium. Albuminuria and glycosuria are found in some cases. In patients where the larynx is primarily affected the most characteristic signs are hoarseness, followed by dyspnœa, which may speedily become urgent; if the epiglottis be infiltrated the patient complains of a "lump in the throat." When the larynx is involved in this disease there is always a risk of sudden death occurring at quite an early stage from œdema, and consequent suffocation. In cases where the larynx becomes secondarily infected from the spread of neighbouring inflammation, especially in such a condition as malignant pustule on the face or neck, the patient may succumb from profound toxæmia rather than from local mechanical causes.

*Treatment.*—This should be very thorough. Careful attention must be paid to any change locally or in the body generally. A conscientious nurse, attentive to details, may easily turn the scales one way or the other. The patient's bed should be in a room at a constant temperature of about 60° F., and it is a good plan to apply an ice-collar round the neck, and to give ice pellets to suck. Administer a calomel purge at the outset in this disease. Calomel is preferable to any other drug for the purpose of evacuating the intestinal contents. Again, every care must be taken to maintain the patient's strength to combat the severe toxæmia, and to this end give by mouth easily assimilable foods, or by rectum if need be. It is well to remember that several eminent physiologists believe that the amount of absorption from the large intestine is very slight. Be prepared to administer stimulants early in this disease. Potass. bromid., 10 to 20 grains, will diminish the tendency to spasm of the glottis; again, ammon. salicylate, 20 grains, given frequently, is often very useful, especially in erysipelatous inflammations associated with high fever, delirium, etc.

It is well worth trying the effect of a polyvalent, anti-streptococcic serum, unless the actual causal organism has been found, in which case that particular organism should be used.

For œdema of larynx a paint of cocaine, 10 to 20 per cent., is most useful, but if this is not successful recourse should be had to scarification, in the manner described in the previous article (February 14, p. 459).

Tracheotomy, generally low in position, must always be in the mind of the surgeon, who should not hesitate to perform the operation if there appears to be any urgency, or the likelihood of it; but here his decision must be guided by the particular circumstances of the case.

It is worth remembering that pilocarpine, $\frac{1}{8}$ grain, given subcutaneously every twenty minutes for three doses, has sometimes brought most rapid and welcome relief to the sufferer, also that inhalations of oxygen might be tried with some hope of success.

6. Acute Œdema.

This specially affects certain parts of the larynx, and, as Tilley rightly points out, it tends to be rather extrinsic than intrinsic; thus it affects the following parts, and generally in this order of priority :

1. Ary-epiglottic folds. Most marked.
2. Glosso-epiglottic fossa. Most marked.
3. Lingual surface of epiglottis. Most marked.
4. Laryngeal surface of epiglottis. Least marked.
5. Vocal cords. Least marked.
6. Sub-glottic region. Least marked.

*Characteristic Symptoms and Signs.*—High temperature. This is generally a marked feature, especially when due to a septic origin. General malaise; much local discomfort, especially a feeling of a "lump in the throat" on swallowing. Dysphagia is generally present, and there is a great increase in the salivary flow; often the patient tends to choke when swallowing fluids, due to imperfect closure of the glottis. Dyspnœa may come on very rapidly, and, indeed, has been known to end fatally in a surprisingly short time from its onset; phonation is impaired, the quality of the voice being profoundly altered; it produces very little resonance, and to the ear of the listener there is imparted a sense of fulness. In later stages the voice may completely disappear.

*Examination.*—The epiglottis is generally erect, and perhaps tense, and swollen to an enormous extent, so that it nearly reaches, in front, to the back of the tongue, and, behind, obscures the ary-epiglottic folds; these latter, when œdematous, as they frequently are in laryngitis of septic origin, may meet in the middle line and thus completely block the airway.

*Treatment.*—(See Acute Septic Laryngitis.)

7. Chronic Laryngitis.

There are at least five types to recognise. viz. :—

(*a*) Simple; (*b*) Chorditis Tuberosa; (*c*) Pachydermia; (*d*) Laryngitis Sicca; and (*e*) Chronic Sub-glottic Laryngitis.

(*a*) Chronic Simple Laryngitis.

*Diagnosis.*—General congestion, dusky-red mucous membrane, the cords having more or less rounded edges, partially obscured by viscid muco-pus; this may not uncommonly form crusts in the inter-arytenoid region. As a consequence the movements of the cords are impaired and phonation is altered in quality.

It is important to note that during an attack of acute pharyngitis the voice, although resembling in some respects that associated with acute laryngitis, shows nevertheless some differences, and this also applies to the chronic stages. In both the resonance is greatly impaired, but in the former the voice is coarser and rougher, and is never lost unless laryngitis be present as well. Ulceration of the cords very rarely occurs in this class of case, but the observer may sometimes notice distended and varicose veins coursing over their surfaces.

The Previous article appeared Feb. 14, p. 459.

**The Severer Forms of Laryngitis**—(*continued*).

Grant states that catarrhal lesions of nose and naso-pharynx may cause a sodden appearance in the mucous membrane of the larynx.

Inter-arytenoid pachydermia is found in a fair number of cases. (See (*c*).)

For the diagnosis from Syphilis, Tubercle, etc., see later.

*Treatment.*—If possible, remove the cause; it is sheer waste of time and trouble and expense to attempt to cure any of these conditions without at first ascertaining the primary origin, and then attacking that; therefore, in every case of laryngitis always examine the nose, naso-pharynx, and mouth. Forbid excess of tobacco, alcohol, and over-use of voice, and, if possible, the patient should learn a few of the elementary principles of natural voice-production. Simple diet, both in fluid and solid, should only be permitted; tea and spirits are harmful; free actions of the bowels and a bracing climate will go far to help on the cure.

Perhaps the most efficient local treatment is a spray of menthol, 20 per cent., in paroleine, used in a de Vilbiss atomiser; it reduces the swelling and loosens the viscid secretion, which is thereby more easily coughed up; again, its antiseptic and anæsthetic properties are not the least valuable of its many good qualities.

The application of other astringents, such as zinc chloride, grains 10 to 1 ounce of water; or ferri perchlor., grains 5 in 1 ounce of water, applied about three times a week, are sometimes helpful. The best method of application of these two drugs is by means of a fine laryngeal spray, adjusted with the help of a laryngoscopic mirror; a previous application of a weak cocaine solution, though not often necessary, will facilitate this application and will greatly diminish the tendency to laryngeal spasm.

